# Obstetric

**Published:** 1881-06

**Authors:** 


### OBSTETRIC.

*Fatal Burns in Advanced Pregnancy. The Burns of the Mother Apparently Impressing Themselves on the Child in Utero.*

This case, reported by Dr. William Hunt, is destined to become classical. A woman, eight and one-half months in pregnancy, received fatal burns from her clothes taking fire, a large part of her body being involved. The fœtal heart-sounds were distinctly heard when the patient was admitted to the hospital, but they ceased during the night, and the next afternoon a well-formed, still-born female child was delivered, healthy, with the exception that its body had the appearance of being recently burned, the epidermis being raised in blisters. Upon comparison, it was found that this appearance of the fœtus corresponded exactly with the mother's burns. Illustrations of the burns of mother and child are furnished, which demonstrate a real resemblance. The bearing of this case upon the question of maternal impressions in the later months of pregnancy is very obvious.—*American Journal of the Medical Sciences for January*, 1881.

